# Gut Microbiome Signatures in Multiple Sclerosis: A Case-Control Study with Machine Learning and Global Data Integration

**DOI:** 10.3390/biomedicines13081806

**Published:** 2025-07-23

**Authors:** Margarita V. Neklesova, Karine S. Sogomonyan, Ivan A. Golovkin, Nikolay I. Shirokiy, Sofia O. Vershinina, Sofia A. Tsvetikova, Julia E. Korzhova, Mariya N. Zakharova, Elena V. Gnedovskaya

**Affiliations:** 1 Institute of Cytology of the Russian Academy of Sciences, 194064 St. Petersburg, Russia; 2 Novabiom, 191119 St. Petersburg, Russia; 3 Faculty of Life Science, ITMO University, 191002 St. Petersburg, Russia; 4 Research Center of Neurology, 125367 Moscow, Russia

**Keywords:** multiple sclerosis, gut microbiota, gut microbiome, 16S rRNA gene sequencing, machine learning, Light Gradient Boosting Machine classifier

## Abstract

**Background/Objectives**: Gut dysbiosis has been implicated in multiple sclerosis (MS), but microbial signatures remain inconsistent across studies. Machine learning (ML) algorithms based on global microbiome data integration can reveal key disease-associated microbial biomarkers and new insights into MS pathogenesis. This study aimed to investigate gut microbial signatures associated with MS and to evaluate the potential of ML for diagnostic applications. **Methods**: Fecal samples from 29 relapsing–remitting MS patients during exacerbation and 27 healthy controls were analyzed using 16S rRNA gene sequencing. Differential abundance analysis was performed, and data were integrated with 29 published studies. Four ML models were developed to distinguish MS-associated microbiome profiles. **Results**: MS patients exhibited reduced levels of Eubacteriales (*p* = 0.037), Lachnospirales (*p* = 0.021), *Oscillospiraceae* (*p* = 0.013), *Lachnospiraceae* (*p* = 0.012), *Parasutterella* (*p* = 0.018), *Faecalibacterium* (*p* = 0.004), and higher abundance of *Lachnospiraceae* UCG-008 (*p* = 0.045) compared to healthy controls. The Light Gradient Boosting Machine classifier demonstrated the highest performance (accuracy: 0.88, AUC-ROC: 0.95) in distinguishing MS microbiome profiles from healthy controls. **Conclusions**: This study highlights specific microbiome dysbiosis in MS patients and supports the potential of ML for diagnostic applications. Further research is needed to elucidate the mechanistic role of these microbial alterations in MS progression and their therapeutic utility.

## 1. Introduction

Multiple sclerosis (MS) is a chronic autoimmune disease of the central nervous system (CNS) characterized by demyelination and neurodegeneration. MS typically manifests between ages 20–40, reduces patients’ quality of life, and leads to permanent disability [1]. It is believed that MS is the result of the interaction of a genetic predisposition and external factors, including sex, viral infections, low vitamin D levels, smoking, and obesity, especially in childhood [2,3].

Growing evidence suggests that gut microbiota may play a critical role in MS pathogenesis by modulating immune and neuroinflammatory pathways. Gut microorganisms regulate the functions of the central nervous system through metabolites such as short-chain fatty acids (SCFAs), vitamins, and neurotransmitters. These substances affect the activity of immune cells, the integrity of the blood–brain barrier (BBB), and the microglial function [4,5,6,7]. Studies on germ-free mice confirm that the absence of microbiota increases BBB permeability [8] and impairs microglial responses to inflammation [9].

MS patients exhibit distinct gut microbial alterations, including reduced levels of SCFA-producing bacteria such as *Prevotella*, *Faecalibacterium*, *Roseburia*, and *Coprococcus*, and increased abundance of potentially pro-inflammatory bacteria like *Akkermansia* and *Blautia* [7,10]. These microbial shifts may contribute to intestinal barrier dysfunction, immune system dysregulation, and neuroinflammation, key factors in the pathogenesis of MS. For instance, butyrate inhibits NF-κB activation and IL-8 production [11] and modulates T-cell differentiation by promoting regulatory T-cell (Treg) formation that suppresses autoimmune reactions [12]. A decrease in *Prevotella* levels, associated with the active phase of MS, leads to reduced production of propionate, a metabolite that inhibits the proliferation of type 17 T helper cells (Th17) capable of penetrating the BBB and exacerbating CNS inflammation [13]. Conversely, elevated *Akkermansia* levels may exacerbate mucosal inflammation due to its mucin-degrading activity [7].

Therapeutic interventions such as dietary modifications, probiotics, and fecal microbiota transplantation (FMT) have shown promising results in modulating the gut microbiome of MS patients, leading to reduced disability, decreased fatigue, improved quality of life, improved metabolic parameters, and lower inflammatory markers [14,15]. Taken together, these findings underscore the significance of the microbiota in MS.

Advances in machine learning (ML) have enabled the identification of disease-specific microbial signatures in conditions such as type 2 diabetes (T2D) [16], inflammatory bowel diseases (IBD) [17], colorectal cancer (CRC) [18], and neurological disorders [19]. Given this potential, we analyzed the gut microbiota of MS patients and healthy controls using both statistical and ML approaches. This strategy not only helps uncover robust microbial biomarkers for MS but also evaluates their utility in early diagnosis, disease monitoring, and personalized treatment strategies.

## 2. Materials and Methods

### 2.1. Study Population

The study included 29 patients with relapsing–remitting type of MS and 27 healthy volunteers as a control group. All participants were recruited from the Research Center of Neurology Clinic (Moscow, Russia) and provided written informed consent. Pseudonymised data from patients with MS and healthy controls were provided to the researchers.

Inclusion criteria for MS patients were (1) age between 18 and 60 years; (2) MS diagnosis confirmed according to the McDonald 2017 criteria [20]; (3) recent clinical relapse onset (<1 month); (4) blood biochemical parameters (glucose and insulin levels, lipid profile, leukocyte formula) within normal ranges. Exclusion criteria for MS patients were (1) glucocorticosteroid therapy within the previous 3 months; (2) antibiotic use within 2 months prior to the study; (3) gastrointestinal diseases (e.g., ulcerative colitis, Crohn’s disease, irritable bowel syndrome) or chronic diseases in active phase; (4) chronic infectious diseases (e.g., tuberculosis, hepatitis B and C, HIV infection); (5) acute infectious diseases such as influenza, within 2 weeks prior to the study; (6) acute intestinal infections within 4 weeks prior to the study; (7) alcohol consumption within 1 week prior to the study; (8) pregnancy or lactation.

Inclusion criteria for healthy volunteers were (1) age between 18 and 60 years; (2) body mass index (BMI) between 18.5 and 27.5 kg/m^2^; (3) blood biochemical parameters (glucose and insulin levels, lipid profile, leukocyte formula) within normal ranges. Exclusion criteria for the control group were (1) use of antibiotics, probiotics, and non-steroidal anti-inflammatory drugs within 3 months prior to the study; (2) diagnosed metabolic diseases (e.g., obesity, atherosclerosis, type 2 diabetes), mental disorders, neurological conditions, oncological diseases, or gastrointestinal diseases (including inflammatory bowel disease or irritable bowel syndrome); (3) chronic infectious diseases (e.g., tuberculosis, hepatitis B and C, HIV infection); (4) acute infectious diseases, such as influenza and acute intestinal infections, within 4 weeks prior to the study; (5) weight loss exceeding 10% of body mass in the previous two months; (6) alcohol consumption within 1 week prior to the study); (7) pregnancy or lactation.

Cohort characteristics were assessed using the Wilcoxon rank-sum test. Categorical variables, such as the presence of specific diseases, were compared using Fisher’s exact test.

### 2.2. Stool Sample Processing and Sequencing

Participants were provided with fecal collection kits (Novabiom, Sirius, Russia). Samples were collected at any time of day without specific dietary restrictions. After collection, samples were transferred to the laboratory at a maintained temperature of −20 °C. Upon receipt, samples were frozen at −20 °C until DNA extraction. Each sample underwent only one freeze–thaw cycle to preserve integrity.

DNA extraction and sequencing were performed at the Cerbalab Genome Center (Saint Petersburg, Russia) following standard protocols. Briefly, bacterial genomic DNA was extracted using the RIBO-prep DNA extraction kit (Cat. K2-9-Et-100; AmpliSens, Moscow, Russia) according to the manufacturer’s instructions. Extracted DNA was stored at −20 °C until PCR amplification. Library preparation was carried out following the standard Illumina protocol (Document #1000000025416 v09) [21]. The V3-V4 regions of the 16S rRNA gene were sequenced with Illumina MiSeq (Illumina, San Diego, CA, USA) using the following primers: forward (5′-TCGTCGGCAGCGTCAGATGTGTATAAGAGACAGCCTACGGGNGGCWGCAG-3′) and reverse (5′-GTCTCGTGGGCTCGGAGATGTGTATAAGAGACAGGACTACHVGGGTATCTAATCC-3′).

### 2.3. Sequence Data Processing

Preprocessing of the raw reads (250 bp, paired-end) was performed using *fastp* [22] with the following parameters: a sliding window size of 5, a mean quality score of 20 per window, and a minimum average quality score of 20 across the read. To assess read quality, trimming was performed from the 3′-end to remove bases with Phred quality scores below 15, corresponding to an accuracy level of 99%. Reads containing more than 15% of positions with a Phred score below 15 were excluded entirely. Additionally, reads shorter than 120 bp after trimming were discarded. These filtering parameters align with commonly accepted practices in 16S rRNA sequencing data analysis. Quality control was performed using FastQC (version 0.11.5) [23] to verify read quality prior to downstream processing. Chimeric reads were removed using the VSEARCH algorithm [24]. Taxonomic classification was performed using the kraken2 algorithm [25] with the SILVA v.138.1 database [26]. Updated taxonomic names were assigned using NCBI taxonomy identifiers for accuracy and consistency with current nomenclature standards. A custom Python (version 3.12.9) script was developed to generate abundance and taxonomy tables at various taxonomic levels.

### 2.4. Statistical Analysis

Data analysis was conducted in R (v4.3.2) using the phyloseq (v1.46.0) [27], microbiome (v1.24.0) [28], and vegan (v2.6-6.1) [29] packages. Taxa marked as “Unknown” and those with zero abundance were excluded to focus on relevant microbial taxa. To filter out low-abundance taxa, thresholds were applied as follows. At the phylum level, taxa with relative abundance below 0.01% were excluded, while for lower-order taxa, those with relative abundance below 0.05% were removed.

#### 2.4.1. Microbial Diversity

α-diversity was analyzed using Chao1, Shannon, and Pielou’s Evenness indices. Statistical significance between groups was assessed with the Wilcoxon test. β-diversity was evaluated using Bray–Curtis distance to quantify dissimilarities between samples. Permutational Multivariate Analysis of Variance (PERMANOVA) was applied to test significant differences in microbial composition between groups.

#### 2.4.2. sPLS-DA

The sparse Partial Least Squares Discriminant Analysis (sPLS-DA) was used to differentiate microbial taxa profiles between individuals with MS and healthy controls, focusing on both classification accuracy and feature selection. The analysis was conducted using the mixOmics package [30]. To account for the compositional nature of microbiome data, a centered log-ratio transformation was applied.

To determine the optimal model complexity, parameter tuning was performed for the number of components and the variables retained on each component. The Balanced Error Rate was used as the evaluation criterion, and five-fold cross-validation with ten repetitions was employed to prevent overfitting and ensure model robustness.

### 2.5. Additional Microbiome Data Collection

A comprehensive literature search was conducted to identify case–control studies utilizing 16S rRNA metagenomic sequencing. The search was performed in the PubMed database using combinations of keywords such as: “gut microbiota; gut microbiome; faecal microbiota; gut bacteria; multiple sclerosis; gut dysbiosis” and “microbial community; gut microbiota; gut microbiome; gut microbiota composition; healthy; body mass index; adults”. Additionally, references from meta-analyses and relevant case–control studies were reviewed to identify additional eligible datasets. Inclusion criteria were established to select studies that provided publicly accessible raw 16S rRNA metagenomic sequencing data and associated metadata clearly indicating case or control status for each sample. The final datasets meeting these criteria are presented in Section 3.5.

### 2.6. Literature Review for Taxonomic Feature Selection

To select features for model training, a comprehensive literature review was conducted to compile taxa that showed significant differences between MS patients and healthy individuals. A search was performed across the PubMed database and employed various keyword combinations including: “intestinal microbiota AND multiple sclerosis”, “gut microbiota AND multiple sclerosis”, and “microbiome signature AND multiple sclerosis”. Selection criteria further emphasized 16S rRNA metagenomic case–control studies comparing MS patients with healthy controls, focusing specifically on bacterial taxa with significant abundance differences between groups.

### 2.7. Development of Machine Learning Models 

The following models were selected for comparison: Random Forest (RF) from scikit-learn [31], eXtreme Gradient Boosting (XGB) (arXiv:1603.02754v3) [32], Light Gradient Boosting Machine (LightGBM), and Support Vector Machine (SVM) from scikit-learn [31]. All models were developed and compared using Python 3. Raw data from the collected studies were processed using a uniform approach described in Section 2.3. Data preprocessing was conducted with Pandas and NumPy Python libraries [33]. Data from open sources were combined with the current study data. To mitigate outliers and address data heterogeneity, the RobustScaler algorithm from scikit-learn fundamental library (allows for implementing machine learning methods in Python) 1.6.1 [31] was employed to normalize the data before model training. For data completeness, we implemented conservative zero-imputation for undetected taxa (NaN → 0). Notably, while LightGBM and XGBoost natively handle missing values, RF and SVM require complete datasets; our zero-imputation made all models applicable within the pipeline. The dataset contained 1705 entities, which were divided into an 80/20 ratio for training and testing, respectively, using a random state of 42 with scikit-learn. Training features were derived from taxa listed in Table S1. Model performance was evaluated using precision, recall, f1-score, accuracy, and accuracy on 5-fold cross-validation using KFold function from scikit-learn [31]. The KFold function implies cross-validation on n-partitioned datasets. A random state of 42 was used during the training.

After comparing the models (Section 3.6), LightGBM was used as the most appropriate due to its advantages: high training speed, efficacy, and efficiency in processing large datasets. Hyperparameter tuning was conducted using Optuna [34] with 100 trials to minimize the value of (1-accuracy). A random state of 48 was set as an independent parameter to ensure result reproducibility. Metrics were calculated using scikit-learn on both the test and train datasets. To evaluate the LightGBM model, precision, recall, accuracy, area under receiver operating characteristic curve (ROC-AUC), and F1-score were calculated, and 5-fold cross-validation for accuracy using KFold was performed. The final LightGBM model parameters are summarized in Table S2.

## 3. Results

### 3.1. Clinical and Demographic Characteristics of the Study Participants

A total of 56 individuals participated in the study, including 29 MS patients and 27 healthy controls. In the MS group, the average disease duration was 4 years. The mean disability level on the Expanded Disability Status Scale (EDSS) was 3. Twenty-two patients were treatment-naïve, while seven had previously used Disease-Modifying Therapies (DMTs). The clinical profile revealed common comorbidities associated with MS, including digestive system issues (e.g., gastritis and constipation) [35], hypertension [36], and overweight/obesity [37]. The demographic characteristics of the groups are summarized in Table 1. Both groups were comparable in terms of age, sex, and other baseline features, except for a significantly higher (*p* < 0.05) prevalence of gastritis in the MS group.

### 3.2. Differences in Gut Microbiota Composition of Healthy Controls and MS Patients

**Figure 1:**
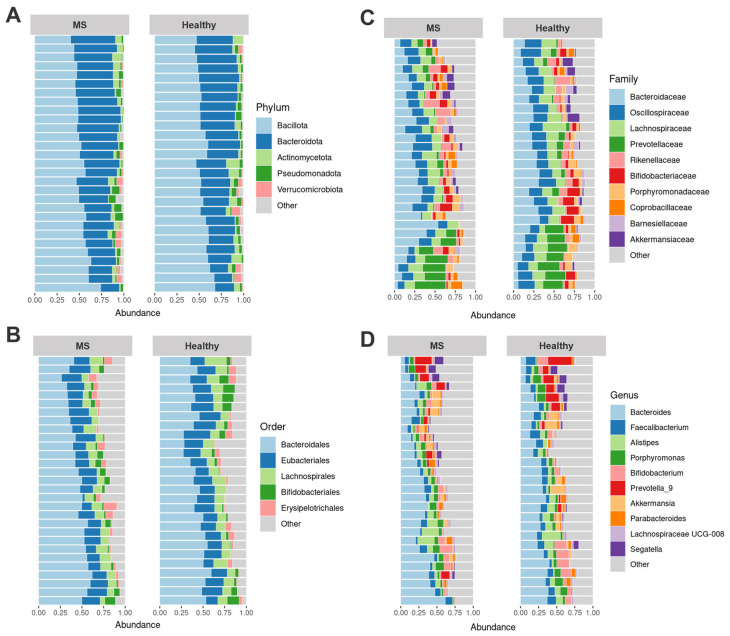
Fecal microbiome composition of healthy controls (27, healthy) and multiple sclerosis (MS) patients (29, MS). The most abundant phyla (**A**), orders (**B**), families (**C**), and genera (**D**) are shown.

The most abundant phyla common to both groups were Bacillota, Bacteroidota, Actinomycetota, Pseudomonadota, and Verrucomicrobiota (Figure 1A). At the order level, prominent taxa included Bacteroidales, Eubacteriales, Lachnospirales, Bifidobacteriales, and Erysipelotrichales (Figure 1B). Dominant families were *Bacteroidaceae*, *Oscillospiraceae*, *Lachnospiraceae*, *Prevotellaceae*, *Rikenellaceae* (Figure 1C). At the genus level, the most abundant taxa were *Bacteroides*, *Faecalibacterium*, *Alistipes*, *Porphyromonas*, *Bifidobacterium*, *Prevotella_9*, *Akkermansia*, *Parabacteroides*, *Lachnospiraceae* UCG-008, and *Segatella* (Figure 1D).

Comparison of the gut microbiota composition between MS patients and healthy controls revealed no significant differences at the phylum level (Table S3). However, MS patients exhibited a lower abundance of Eubacteriales (*p* = 0.037), Lachnospirales (*p* = 0.021), *Oscillospiraceae* (*p* = 0.013), *Lachnospiraceae* (*p* = 0.012), *Parasutterella* (*p* = 0.018), *Faecalibacterium* (*p* = 0.004), and higher abundance of *Lachnospiraceae* UCG-008 (*p* = 0.045) compared to healthy controls.

### 3.3. Bacterial Diversity Analysis

To evaluate overall differences in microbial community structure between MS patients and healthy controls, α-diversity and β-diversity measures were calculated. α-diversity analysis, which assesses richness and evenness at the phylum, order, family, and genus levels, showed no significant differences in microbial diversity between MS patients and healthy controls (Table S4). β-diversity analysis, which examines dissimilarities in microbial community composition, also revealed no discernible clustering trends between the two groups at any taxonomic level (PERMANOVA, *p*-values: 0.265, 0.131, 0.206, and 0.228 for phylum, order, family, and genus levels, respectively).

### 3.4. sPLS-DA Analysis Results

**Figure 2:**
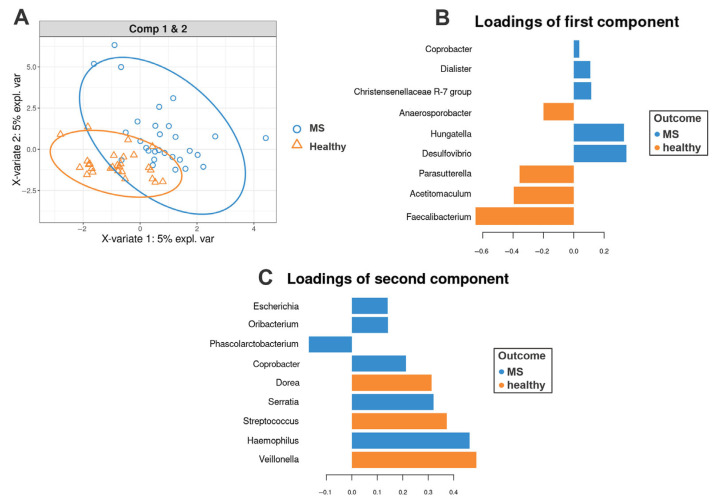
sPLS-DA of the gut microbiota in multiple sclerosis (MS) patients and healthy controls. (**A**) sPLS-DA score plot illustrating the separation between healthy controls and MS patients based on gut microbiota composition at the genus level. Ellipses represent 95% confidence intervals for each group. (**B**,**C**) show the most discriminative bacterial genera for components 1 and 2, respectively. Genera are ranked from bottom to top according to their contribution to the corresponding component. Loading weights for healthy controls are shown in orange, while those for MS patients are depicted in blue.

The sPLS-DA analysis demonstrated separation between MS and healthy controls, identifying key taxa contributing to group differences (Figures S1–S3). The following genera (Figure 2B,C) contributed (|loading vectors| > 0.25) to the first and second components of the sPLS-DA: *Hungatella*, *Desulfovibrio*, *Acetitomaculuum*, *Parasutterella*, *Faecalibacterium*, *Dorea*, *Serratia*, *Streptococcus*, *Haemophilus*, *Veillonella*. Notably, these results align with the findings from the comparison of taxa relative abundance (Section 3.2): MS patients also exhibited a significantly reduced relative abundance of *Parasutterella* (*p* = 0.018) and *Faecalibacterium* (*p* = 0.004) compared to healthy controls.

### 3.5. Dataset for the Global Analysis

The initial sample of 56 individuals is insufficient for ML algorithms to effectively distinguish between the gut microbiota of MS patients and healthy controls. To address this limitation, we conducted an extensive literature review and integrated publicly available data. Details of the literature search strategy are provided in Section 2.5.

The final dataset included 29 studies (Table 2). Patient metadata was filtered based on the following criteria:

- For MS patients: Age ≤ 70 years, BMI ≤ 36 kg/m^2^, and no antibiotic use for at least one month prior to fecal sample collection.
- For healthy volunteers: Age ≤ 50 years, BMI between 18.5 and 25 kg/m^2^, and the same antibiotic restriction.

These filtered samples were supplemented with data from our cohort. The resulting sample sizes were 777 for healthy controls and 928 for MS patients.

### 3.6. Development of an ML Algorithm for Microbiome Classification

To identify the most relevant features for model training, an extensive literature analysis was conducted. The detailed search strategy is described in Section 2.6. This literature-based list of bacteria associated with MS was supplemented with taxa that demonstrated significant differences in the present study (Section 3.2). This integrated feature selection approach, combining evidence from both published literature and experimental data from this study, resulted in a final selection of thirty-six taxa (Table S1).

The training dataset consisted of 1363 samples: 750 MS patients and 613 healthy controls, and the test dataset included 342 samples: 178 MS patients and 164 healthy controls. Table 3 presents the comparative performance metrics of four ML models on the test dataset. Evaluation metrics include precision, recall, F1-score, accuracy, and cross-validation accuracy.

Based on the comparison (Table 3), XGB and LightGBM show similar classification performance, but LightGBM achieves higher metrics for cross-validation. Moreover, LightGBM trains faster and has higher efficacy in processing larger datasets [67]. Therefore, LightGBM was selected as the primary model for microbiome classification. Further optimization was performed using the Optuna tool, and its final predictive performance is presented in Table 4.

**Figure 3:**
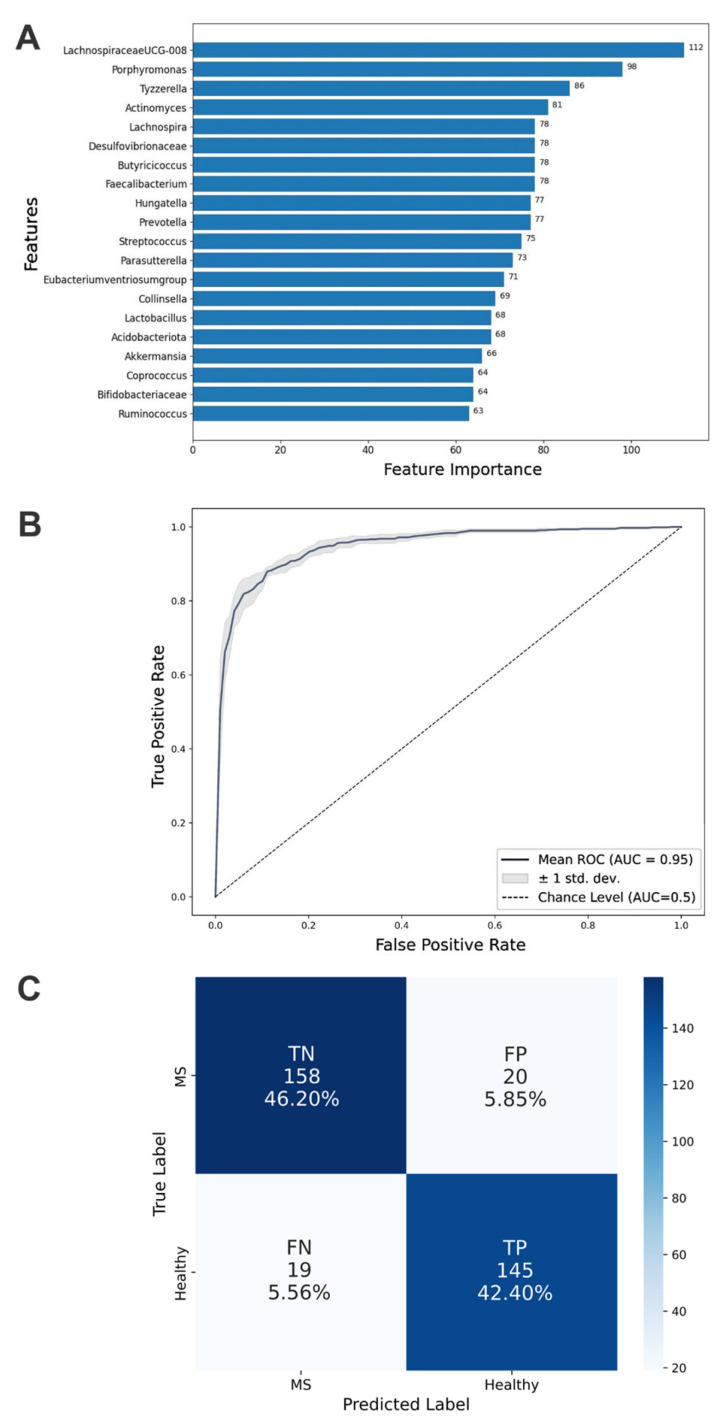
Aspects of classification performance achieved by the optimized Light Gradient Boosting Machine model. (**A**) Top 20 important taxa identified by feature importance scores. These taxa are crucial for the model’s classification capability and demonstrate each microbial factor’s relative contribution to distinguishing multiple sclerosis states. (**B**) Mean AUC (Area Under Curve) of the ROC (Receiver Operating Characteristic) curve for the test dataset from 5-fold cross-validation. (**C**) Confusion matrix detailing true positive (TP), true negative (TN), false positive (FP), and false negative (FN) counts for the classification of MS patients and healthy controls.

Values ranging from 0.86 to 0.91 across all metrics indicate strong model performance (Table 4). The model effectively detects positive cases (recall = 0.84) while maintaining accurate predictions (precision = 0.9), demonstrating a balanced and reliable classification capability. Feature importance analysis revealed that *Lachnospiraceae* UCG-008 and *Porphyromonas* were the most critical taxa for health state classification, with other taxa contributing proportionally (Figure 3A).

The ROC curve provides a graphical representation of diagnostic performance across various thresholds, with a higher AUC indicating better predictive performance and generalizability to unseen data. The optimized LightGBM model achieved an AUC-ROC score of 0.95, highlighting its exceptional ability to rank samples correctly (Figure 3B). This suggests that the model ranks positive examples higher than negative ones in sorted probability lists.

To analyze prediction errors, a confusion matrix was constructed (Figure 3C). The matrix reveals symmetrical patterns of misclassification, indicating that the LightGBM model exhibits a comparable error rate when incorrectly classifying healthy individuals as having MS and when misclassifying MS patients as healthy. This balanced distribution of errors suggests that the classifier does not show a systematic bias toward either false positives or false negatives.

## 4. Discussion

This study examined gut microbiota changes in relapsing–remitting MS patients during exacerbation compared with healthy controls. By focusing on the active disease phase, we minimized disease activity heterogeneity, as prior evidence indicates distinct microbiota dynamics during relapse versus remission [13,47].

Consistent with large-scale MS studies [11,38], we observed no intergroup differences in α-diversity. Notably, β-diversity analysis showed no significant differences in our predominantly DMT-naïve cohort from controls, aligning with findings from the cohort in [68], where no significant differences were found between untreated MS patients and healthy controls. This contrasts with reports of β-diversity alterations in mixed groups, including both treated and untreated patients [11,38]. Potential explanations for this discrepancy include (1) our smaller sample size; (2) regional variations in microbiota composition influenced by geographic, dietary, and environmental factors; and (3) the treatment-naïve status of most participants, as DMTs are known to influence gut microbiota composition [42].

Despite preserved overall microbial community structure, relative abundance analysis identified seven differentially abundant taxa, with *Faecalibacterium* and *Parasutterella* emerging as key discriminators in sPLS-DA analysis. *Parasutterella* converts primary bile acids (e.g., cholic acid) into secondary forms, modulating FXR-mediated anti-inflammatory signaling [69]. Its depletion in this study contrasts with reports of increased *Parasutterella* abundance in other MS [70,71] and Crohn’s disease studies [72], possibly due to differences in disease stage, treatment, or microbial niche (luminal or mucosal). Reduced *Parasutterella* levels may impair bile acid metabolism, weakening FXR signaling and exacerbating neuroinflammation. Additionally, strain-specific variability could influence these effects. The exact role of *Parasutterella* in MS remains unclear, highlighting the need for longitudinal studies accounting for disease progression and therapy.

A decreased relative abundance of *Lachnospiraceae* and *Oscillospiraceae* in MS patients warrants particular attention, since these families include key producers of SCFAs such as *Faecalibacterium*, *Roseburia*, *Anaerostipes*, *Coprococcus*, and *Lachnospira*, which play an important role in maintaining the integrity of the intestinal barrier and immune modulation [7,11,73,74]. Butyrate, a key SCFA produced by these taxa, serves as an energy source for colonocytes and strengthens the intestinal barrier by stimulating mucin synthesis and expression of intestinal epithelial tight junctions’ proteins, as well as suppressing pro-inflammatory reactions through inhibition of NF-kB and histone deacetylases [75,76]. Moreover, SCFAs, especially butyrate and propionate, regulate the immune response through their effect on Tregs, which maintain tolerance to their own antigens and prevent autoimmune reactions. Propionate and acetate promote the accumulation of Tregs in the large intestine, while butyrate and propionate enhance their differentiation [77]. This is consistent with data from other studies demonstrating a decrease in the content of butyrate-producing bacteria (*Roseburia*, *Anaerostipes*, *Coprococcus*) in MS [7,73,74], as well as with the hypothesis that SCFAs deficiency exacerbates neuroinflammation.

The observed reduction in *Faecalibacterium*, noted in this study, is of particular significance, since its depletion has been associated not only with MS, but also with T2D [78], depression [79], obesity [80], and irritable bowel syndrome [81], which emphasizes its central role in maintaining immune and metabolic balance. Thus, a decrease in SCFAs production with a decrease in the number of *Lachnospiraceae*, *Oscillospiraceae*, and *Faecalibacterium* can not only weaken the intestinal barrier but also disrupt the regulation of the immune response, contributing to the autoimmune inflammation in MS.

Conversely, the increased prevalence of *Lachnospiraceae* UCG-008 in MS patients is notable. In spinal cord injury patients, *Lachnospiraceae* UCG-008 negatively correlates with CD3+ T-cells [82], indicating a likely ability to suppress T-cell proliferation or function, which may exacerbate immune dysfunction. A similar increase in *Lachnospiraceae* UCG-008 has been reported in CRC patients [83], further supporting its possible involvement in immune modulation. In MS, such mechanisms might involve T-cell modulation, where the inhibition of CD3+ T-cells could disrupt the Th17/Treg balance, skewing toward pro-inflammatory responses. Alternatively, the increase in *Lachnospiraceae* UCG-008 might also represent a compensatory response to chronic inflammation, though insufficient to restore homeostasis in MS. Further research is needed to clarify whether *Lachnospiraceae* UCG-008 actively contributes to MS pathogenesis or emerges as a bystander in inflammation.

ML models comparison revealed that the LightGBM outperformed XGB, RF, and SVM, achieving high accuracy (0.88), F1-score (0.86), and AUC-ROC (0.95). These metrics correspond to or exceed the benchmarks in microbiome-disease studies (e.g., AUC-ROC 0.84–0.95 for CRC, IBD, and T2D [84,85,86], highlighting the microbiome’s potential as a diagnostic biomarker for MS.

Collectively, our results highlight that despite preserved global microbial diversity, MS is associated with distinct alterations in specific taxa, particularly those involved in SCFAs production (e.g., *Faecalibacterium*, *Oscillospiraceae*, *Lachnospiraceae*) and bile acid metabolism (*Parasutterella*). The functional consequences of these shifts—including reduced SCFAs synthesis, compromised intestinal barrier integrity, and dysregulated immune signaling—may collectively contribute to neuroinflammation in MS. The robust discriminatory power of LightGBM underscores the potential of microbiome-based diagnostic approaches for MS. However, the generalizability of these microbial signatures across diverse cohorts requires further validation.

Several limitations should be acknowledged. Firstly, the cross-sectional design precludes causal inferences about whether the observed microbiome alterations contribute to MS pathogenesis or result from disease processes. Additionally, confounding variables (e.g., diet, lifestyle, DMTs, comorbidities) could influence both microbiota composition, further complicating causal interpretation. Finally, the sample size may limit the detection of subtle microbial changes. Future work should prioritize a meta-analysis of specific bacterial taxa—quantifying effect sizes, consistency across studies, and potential mechanisms. Such an approach would help distinguish robust microbial associations from context-dependent findings and clarify their relevance to MS pathogenesis. Longitudinal studies tracking microbiome dynamics from pre-symptomatic stages, combined with mechanistic investigations in experimental models, will also be crucial to establish directionality and therapeutic relevance of these associations.

## 5. Conclusions

This study identified key differences in gut microbiome composition between patients with MS during exacerbation and healthy controls. While α- and β-diversity metrics showed no significant differences between the groups, specific alterations in bacterial taxa were observed, including a reduction in SCFA-producing bacteria (*Faecalibacterium*, *Oscillospiraceae*, *Lachnospiraceae*) and decreased *Parasutterella*, which is involved in bile acid metabolism. The LightGBM classifier demonstrated high diagnostic accuracy (AUC-ROC of 0.95), supporting the potential of microbiome-based biomarkers for MS. However, study limitations—including sample size and cross-sectional design—preclude causal inferences. Future longitudinal and mechanistic studies are needed to determine whether these microbial changes drive neuroinflammation or are secondary to disease progression.

## Figures and Tables

**Table 1:** Clinical and demographic features of multiple sclerosis (MS) patients and the control group.

Characteristics	MS (n = 29)	Healthy (n = 27)	*p*-Value
Age (years) *	33.9 ± 11.1 (19–60 years)	31.0 ± 10.8 (19–60 years)	0.34 ^a^
Sex (M/F)	(13/16)	(11/16)	1 ^b^
BMI, kg/m^2^ *	24.8 ± 5.9 (18.6–46.9)	21.9 ± 2.5 (18.5–27.7)	0.051 ^a^
Gastritis, n (%)	10 (34.5)	0 (0)	0.001 ^c^
Constipation, n (%)	5 (17.2)	0 (0)	0.052 ^c^
Overweight (BMI 25–29.9 kg/m^2^), n (%)	6 (20.7)	1 (3.7)	0.1 ^c^
Obesity (BMI > 30 kg/m^2^), n (%)	4 (13.8)	0 (0)	0.11 ^c^
Hypertension, n (%)	3 (10.3)	0 (0)	0.24 ^c^

Statistical tests: ^a^ Wilcoxon rank-sum test. ^b^ Chi-squared test. ^c^ Fisher’s exact test. * Average ± SD, (min–max).

**Table 2:** Summary of datasets collected and filtered for development of machine learning models.

Dataset ID	Country	N (Controls)	N (Cases)	Reference
Current study	Russia	27	29	-
Cox L. M. et al., 2021	USA	15	327	[38]
Takewaki D. et al., 2022	Japan	-	98	[39]
Elsayed N. S. et al., 2023	USA	-	83	[40]
Kozhieva M. et al., 2021	Russia	-	63	[41]
Jangi S. et al., 2016	USA	-	60	[42]
Cekanaviciute E. et al., 2017	USA	-	57	[43]
Forbes J. D. et al., 2018	Canada	-	40	[44]
Saresella M. et al., 2020	Italy	-	35	[45]
Zeng Q. et al., 2019	China	-	30	[46]
Chen J. et al., 2016	USA	-	30	[47]
Kozhieva M. et al., 2019	Russia	-	15	[48]
Barone M. et al., 2021	Italy	-	15	[49]
Ascanelli S. et al., 2022	Italy	-	12	[50]
Yadav S. K. et al., 2022	USA	13	13	[51]
Ling Z. et al., 2020	China	18	21	[52]
Gallè F. et al., 2020	Italy	107	-	[53]
Park J. et al., 2021	Japan	92	-	[54]
Su Q. et al., 2023	China	90	-	[55]
Healey G. et al., 2018	New Zealand	75	-	[56]
De La Cuesta-Zuluaga J. et al., 2018	Colombia	63	-	[57]
Bailén M. et al., 2020	Spain	52	-	[58]
Dhakan D. B. et al., 2019	India	49	-	[59]
Serrano J. et al., 2021	USA	44	-	[60]
Wang T. et al., 2022	Netherlands	33	-	[61]
Li H. et al., 2021	China	30	-	[62]
Mancabelli L. et al., 2017	Italy	23	-	[63]
Rodriguez J. et al., 2020	Belgium	20	-	[64]
Borgo F. et al., 2018	Italy	10	-	[65]
Gaike A. H. et al., 2020	India	16	-	[66]

**Table 3:** Machine learning (ML) models comparison in prediction tasks on the test dataset.

Metric	ML Model			
	RF	XGB	SVM	LightGBM
Precision	0.88	0.89	0.83	0.90
Recall	0.82	0.87	0.48	0.84
F1-score	0.85	0.88	0.60	0.87
Accuracy	0.86	0.89	0.70	0.88
Cross-validation accuracy	0.84	0.85	0.69	0.87

**Table 4:** Optimized Light Gradient Boosting Machine model classification performances on the test dataset. Classification metrics include accuracy, precision, recall, and F1-score.

	Precision	Recall	F1-Score
Healthy controls	0.91	0.86	0.88
MS patients	0.89	0.92	0.91

## Data Availability

The data that support the findings of this study are available from the corresponding author upon reasonable request.

## Supplementary Materials

The following supporting information can be downloaded at https://www.mdpi.com/article/10.3390/biomedicines13081806/s1. Figure S1: sPLS-DA of the gut microbiota in multiple sclerosis (MS) patients and healthy controls at the phylum level. (A) sPLS-DA score plot illustrating the separation between healthy controls and MS patients based on gut microbiota composition. Ellipses represent 95% confidence intervals for each group. (B,C) show the most discriminative phyla for components 1 and 2, respectively. Taxa are ranked from bottom to top according to their contribution to the corresponding component. Loading weights for healthy controls are shown in orange, while those for MS patients are depicted in blue; Figure S2: sPLS-DA of the gut microbiota in multiple sclerosis (MS) patients and healthy controls at the order level. (A) sPLS-DA score plot illustrating the separation between healthy controls and MS patients based on gut microbiota composition. Ellipses represent 95% confidence intervals for each group. (B,C) show the most discriminative orders for components 1 and 2, respectively. Taxa are ranked from bottom to top according to their contribution to the corresponding component. Loading weights for healthy controls are shown in orange, while those for MS patients are depicted in blue; Figure S3: sPLS-DA of the gut microbiota in multiple sclerosis (MS) patients and healthy controls at the family level. (A) sPLS-DA score plot illustrating the separation between healthy controls and MS patients based on gut microbiota composition. Ellipses represent 95% confidence intervals for each group. (B,C) show the most discriminative families for components 1 and 2, respectively. Taxa are ranked from bottom to top according to their contribution to the corresponding component. Loading weights for healthy controls are shown in orange, while those for MS patients are depicted in blue; Table S1: Taxa selected for model training as significant for multiple sclerosis (MS). Based on case–control MS studies and results from our study; Table S2: Light Gradient Boosting Machine model hyperparameters; Table S3: Compositional differences in major taxa in the gut microbiota of multiple sclerosis (MS) patients and healthy controls. The analysis was carried out using the Wilcoxon criterion, with significant differences in taxa highlighted in bold; Table S4: α-diversity analysis (Chao1, Shannon, Pielou indexes) of gut microbiota of multiple sclerosis (MS) patients and healthy controls. Statistical significance was assessed using the Wilcoxon test. References [87,88,89,90,91,92,93,94] are cited in Supplementary Materials.

## Abbreviations

The following abbreviations are used in this manuscript:

AUC	Area Under Curve
BMI	Body Mass Index
CNS	Central Nervous System
CRC	Colorectal Cancer
DMTs	Disease-Modifying Therapies
EDSS	Expanded Disability Status Scale
IBD	Inflammatory Bowel Diseases
LightGBM	Light Gradient Boosting Machine
ML	Machine Learning
MS	Multiple Sclerosis
RF	Random Forest
ROC	Receiver Operating Characteristic
SCFAs	Short-Chain Fatty Acids
sPLS-DA	Sparse Partial Least Squares Discriminant Analysis
SVM	Support Vector Machine
T2D	Type 2 Diabetes
Tregs	Regulatory T-cells
XGB	eXtreme Gradient Boosting

## References

[1] Yamout B., Sahraian M., Bohlega S., Al-Jumah M., Goueider R., Dahdaleh M., Inshasi J., Hashem S., Alsharoqi I., Khoury S. (2020). Consensus recommendations for the diagnosis and treatment of multiple sclerosis: 2019 revisions to the MENACTRIMS guidelines. Mult. Scler. Relat. Disord..

[2] Olsson T., Barcellos L.F., Alfredsson L. (2017). Interactions between genetic, lifestyle and environmental risk factors for multiple sclerosis. Nat. Rev. Neurol..

[3] Ramagopalan S.V., Dobson R., Meier U.C., Giovannoni G. (2010). Multiple sclerosis: Risk factors, prodromes, and potential causal pathways. Lancet Neurol..

[4] Chu F., Shi M., Lang Y., Shen D., Jin T., Zhu J., Cui L. (2018). Gut Microbiota in Multiple Sclerosis and Experimental Autoimmune Encephalomyelitis: Current Applications and Future Perspectives. Mediat. Inflamm..

[5] Calvo-Barreiro L., Eixarch H., Montalban X., Espejo C. (2018). Combined therapies to treat complex diseases: The role of the gut microbiota in multiple sclerosis. Autoimmun. Rev..

[6] Abdel-Haq R., Schlachetzki J.C.M., Glass C.K., Mazmanian S.K. (2019). Microbiome-microglia connections via the gut-brain axis. J. Exp. Med..

[7] Ordoñez-Rodriguez A., Roman P., Rueda-Ruzafa L., Campos-Rios A., Cardona D. (2023). Changes in Gut Microbiota and Multiple Sclerosis: A Systematic Review. Int. J. Environ. Res. Public Health.

[8] Braniste V., Al-Asmakh M., Kowal C., Anuar F., Abbaspour A., Tóth M., Korecka A., Bakocevic N., Ng L.G., Kundu P. (2014). The gut microbiota influences blood-brain barrier permeability in mice. Sci. Transl. Med..

[9] Erny D., Hrabě de Angelis A.L., Jaitin D., Wieghofer P., Staszewski O., David E., Keren-Shaul H., Mahlakoiv T., Jakobshagen K., Buch T. (2015). Host microbiota constantly control maturation and function of microglia in the CNS. Nat. Neurosci..

[10] Fettig N.M., Osborne L.C. (2021). Direct and indirect effects of microbiota-derived metabolites on neuroinflammation in multiple sclerosis. Microbes Infect..

[11] Zhou X., Baumann R., Gao X., Mendoza M., Singh S., Sand I.K., Xia Z., Cox L.M., Chitnis T., Yoon H. (2022). Gut microbiome of multiple sclerosis patients and paired household healthy controls reveal associations with disease risk and course. Cell.

[12] Ríos-Covián D., Ruas-Madiedo P., Margolles A., Gueimonde M., de Los Reyes-Gavilán C.G., Salazar N. (2016). Intestinal Short Chain Fatty Acids and their Link with Diet and Human Health. Front. Microbiol..

[13] Cosorich I., Dalla-Costa G., Sorini C., Ferrarese R., Messina M.J., Dolpady J., Radice E., Mariani A., Testoni P.A., Canducci F. (2017). High frequency of intestinal T_H_17 cells correlates with microbiota alterations and disease activity in multiple sclerosis. Sci. Adv..

[14] Bronzini M., Maglione A., Rosso R., Matta M., Masuzzo F., Rolla S., Clerico M. (2023). Feeding the gut microbiome: Impact on multiple sclerosis. Front. Immunol..

[15] Tsogka A., Kitsos D.K., Stavrogianni K., Giannopapas V., Chasiotis A., Christouli N., Tsivgoulis G., Tzartos J.S., Giannopoulos S. (2023). Modulating the Gut Microbiome in Multiple Sclerosis Management: A Systematic Review of Current Interventions. J. Clin. Med..

[16] Kavakiotis I., Tsave O., Salifoglou A., Maglaveras N., Vlahavas I., Chouvarda I. (2017). Machine Learning and Data Mining Methods in Diabetes Research. Comput. Struct. Biotechnol. J..

[17] Gubatan J., Levitte S., Patel A., Balabanis T., Wei M.T., Sinha S.R. (2021). Artificial intelligence applications in inflammatory bowel disease: Emerging technologies and future directions. World J. Gastroenterol..

[18] Thomas A.M., Manghi P., Asnicar F., Pasolli E., Armanini F., Zolfo M., Beghini F., Manara S., Karcher N., Pozzi C. (2019). Metagenomic analysis of colorectal cancer datasets identifies cross-cohort microbial diagnostic signatures and a link with choline degradation. Nat. Med..

[19] Pietrucci D., Teofani A., Unida V., Cerroni R., Biocca S., Stefani A., Desideri A. (2020). Can Gut Microbiota Be a Good Predictor for Parkinson’s Disease? A Machine Learning Approach. Brain Sci..

[20] Thompson A.J., Banwell B.L., Barkhof F., Carroll W.M., Coetzee T., Comi G., Correale J., Fazekas F., Filippi M., Freedman M.S. (2018). Diagnosis of multiple sclerosis: 2017 revisions of the McDonald criteria. Lancet Neurol..

[21] (2020). Illumina DNA Prep Reference Guide—Document #1000000025416, v09.

[22] Chen S., Zhou Y., Chen Y., Gu J. (2018). fastp: An ultra-fast all-in-one FASTQ preprocessor. Bioinformatics.

[23] Andrews S. (2010). FastQC: A Quality Control Tool for High Throughput Sequence Data. http://www.bioinformatics.babraham.ac.uk/projects/fastqc/.

[24] Rognes T., Flouri T., Nichols B., Quince C., Mahé F. (2016). VSEARCH: A versatile open source tool for metagenomics. PeerJ.

[25] Wood D.E., Lu J., Langmead B. (2019). Improved metagenomic analysis with Kraken 2. Genome Biol..

[26] Quast C., Pruesse E., Yilmaz P., Gerken J., Schweer T., Yarza P., Peplies J., Glöckner F.O. (2012). The SILVA ribosomal RNA gene database project: Improved data processing and web-based tools. Nucleic Acids Res..

[27] McMurdie P.J., Holmes S. (2013). phyloseq: An R Package for Reproducible Interactive Analysis and Graphics of Microbiome Census Data. PLoS ONE.

[28] Lahti L., Shetty S. (2017). Tools for Microbiome Analysis in R Version. https://github.com/microbiome/microbiome.

[29] Oksanen J., Kindt R., Legendre P. (2007). The Vegan Package. Community Ecol. Package.

[30] Rohart F., Gautier B., Singh A., Lê Cao K.-A. (2017). mixOmics: An R package for ‘omics feature selection and multiple data integration. PLoS Comput. Biol..

[31] Pedregosa F., Varoquaux G., Gramfort A., Michel V., Thirion B., Grisel O., Blondel M., Prettenhofer P., Weiss R., Dubourg V. (2011). Scikit-learn: Machine learning in Python. J. Mach. Learn. Res..

[32] Chen T., Guestrin C. (2016). XGBoost: A Scalable Tree Boosting System. arXiv.

[33] Harris C.R., Millman K.J., van der Walt S.J., Gommers R., Virtanen P., Cournapeau D., Wieser E., Taylor J., Berg S., Smith N.J. (2020). Array programming with NumPy. Nature.

[34] Akiba T., Sano S., Yanase T., Ohta T., Koyama M. Optuna: A Next-generation Hyperparameter Optimization Framework. Proceedings of the 25th ACM SIGKDD International Conference on Knowledge Discovery & Data Mining.

[35] Preziosi G., Gordon-Dixon A., Emmanuel A. (2018). Neurogenic bowel dysfunction in patients with multiple sclerosis: Prevalence, impact, and management strategies. Degener. Neurol. Neuromuscul. Dis..

[36] Marrie R.A., Rudick R., Horwitz R., Cutter G., Tyry T., Campagnolo D., Vollmer T. (2010). Vascular comorbidity is associated with more rapid disability progression in multiple sclerosis. Neurology.

[37] Marck C.H., Neate S.L., Taylor K.L., Weiland T.J., Jelinek G.A. (2016). Prevalence of Comorbidities, Overweight and Obesity in an International Sample of People with Multiple Sclerosis and Associations with Modifiable Lifestyle Factors. PLoS ONE.

[38] Cox L.M., Maghzi A.H., Liu S., Tankou S.K., Dhang F.H., Willocq V., Song A., Wasén C., Tauhid S., Chu R. (2021). Gut Microbiome in Progressive Multiple Sclerosis. Ann. Neurol..

[39] Takewaki D., Suda W., Sato W., Takayasu L., Kumar N., Kimura K., Kaga N., Mizuno T., Miyake S., Hattori M. (2020). Alterations of the gut ecological and functional microenvironment in different stages of multiple sclerosis. Proc. Natl. Acad. Sci. USA.

[40] Elsayed N.S., Valenzuela R.K., Kitchner T., Le T., Mayer J., Tang Z.Z., Bayanagari V.R., Lu Q., Aston P., Anantharaman K. (2023). Genetic risk score in multiple sclerosis is associated with unique gut microbiome. Sci. Rep..

[41] Kozhieva M., Naumova N., Alikina T., Boyko A., Vlassov V., Kabilov M.R. (2021). The Core of Gut Life: Firmicutes Profile in Patients with Relapsing-Remitting Multiple Sclerosis. Life.

[42] Jangi S., Gandhi R., Cox L.M., Li N., von Glehn F., Yan R., Patel B., Mazzola M.A., Liu S., Glanz B.L. (2016). Alterations of the human gut microbiome in multiple sclerosis. Nat. Commun..

[43] Cekanaviciute E., Yoo B.B., Runia T.F., Debelius J.W., Singh S., Nelson C.A., Kanner R., Bencosme Y., Lee Y.K., Hauser S.L. (2017). Gut bacteria from multiple sclerosis patients modulate human T cells and exacerbate symptoms in mouse models. Proc. Natl. Acad. Sci. USA.

[44] Forbes J.D., Chen C.Y., Knox N.C., Marrie R.A., El-Gabalawy H., de Kievit T., Alfa M., Bernstein C.N., Van Domselaar G. (2018). A comparative study of the gut microbiota in immune-mediated inflammatory diseases—Does a common dysbiosis exist?. Microbiome.

[45] Saresella M., Marventano I., Barone M., La Rosa F., Piancone F., Mendozzi L., d’Arma A., Rossi V., Pugnetti L., Roda G. (2020). Alterations in Circulating Fatty Acid Are Associated with Gut Microbiota Dysbiosis and Inflammation in Multiple Sclerosis. Front. Immunol..

[46] Zeng Q., Gong J., Liu X., Chen C., Sun X., Li H., Zhou Y., Cui C., Wang Y., Yang Y. (2019). Gut dysbiosis and lack of short chain fatty acids in a Chinese cohort of patients with multiple sclerosis. Neurochem. Int..

[47] Chen J., Chia N., Kalari K.R., Yao J.Z., Novotna M., Paz Soldan M.M., Luckey D.H., Marietta E.V., Jeraldo P.R., Chen X. (2016). Multiple sclerosis patients have a distinct gut microbiota compared to healthy controls. Sci. Rep..

[48] Kozhieva M., Naumova N., Alikina T., Boyko A., Vlassov V., Kabilov M.R. (2019). Primary progressive multiple sclerosis in a Russian cohort: Relationship with gut bacterial diversity. BMC Microbiol..

[49] Barone M., Mendozzi L., D’Amico F., Saresella M., Rampelli S., Piancone F., La Rosa F., Marventano I., Clerici M., d’Arma A. (2021). Influence of a High-Impact Multidimensional Rehabilitation Program on the Gut Microbiota of Patients with Multiple Sclerosis. Int. J. Mol. Sci..

[50] Ascanelli S., Bombardini C., Chimisso L., Carcoforo P., Turroni S., D’Amico F., Caniati M.L., Baldi E., Tugnoli V., Morotti C. (2022). Trans-anal irrigation in patients with multiple sclerosis: Efficacy in treating disease-related bowel dysfunctions and impact on the gut microbiota: A monocentric prospective study. Mult. Scler. J. Exp. Transl. Clin..

[51] Yadav S.K., Ito N., Mindur J.E., Kumar H., Youssef M., Suresh S., Kulkarni R., Rosario Y., Balashov K.E., Dhib-Jalbut S. (2022). Fecal Lcn-2 level is a sensitive biological indicator for gut dysbiosis and intestinal inflammation in multiple sclerosis. Front. Immunol..

[52] Ling Z., Cheng Y., Yan X., Shao L., Liu X., Zhou D., Zhang L., Yu K., Zhao L. (2020). Alterations of the Fecal Microbiota in Chinese Patients With Multiple Sclerosis. Front. Immunol..

[53] Gallè F., Valeriani F., Cattaruzza M.S., Gianfranceschi G., Liguori R., Antinozzi M., Mederer B., Liguori G., Romano Spica V. (2020). Mediterranean Diet, Physical Activity and Gut Microbiome Composition: A Cross-Sectional Study among Healthy Young Italian Adults. Nutrients.

[54] Park J., Kato K., Murakami H., Hosomi K., Tanisawa K., Nakagata T., Ohno H., Konishi K., Kawashima H., Chen Y.A. (2021). Comprehensive analysis of gut microbiota of a healthy population and covariates affecting microbial variation in two large Japanese cohorts. BMC Microbiol..

[55] Su Q., Tun H.M., Liu Q., Yeoh Y.K., Mak J.W.Y., Chan F.K., Ng S.C. (2023). Gut microbiome signatures reflect different subtypes of irritable bowel syndrome. Gut Microbes.

[56] Healey G., Murphy R., Butts C., Brough L., Whelan K., Coad J. (2018). Habitual dietary fibre intake influences gut microbiota response to an inulin-type fructan prebiotic: A randomised, double-blind, placebo-controlled, cross-over, human intervention study. Br. J. Nutr..

[57] de la Cuesta-Zuluaga J., Corrales-Agudelo V., Carmona J.A., Abad J.M., Escobar J.S. (2018). Body size phenotypes comprehensively assess cardiometabolic risk and refine the association between obesity and gut microbiota. Int. J. Obes..

[58] Bailén M., Bressa C., Martínez-López S., González-Soltero R., Montalvo Lominchar M.G., San Juan C., Larrosa M. (2020). Microbiota Features Associated with a High-Fat/Low-Fiber Diet in Healthy Adults. Front. Nutr..

[59] Dhakan D.B., Maji A., Sharma A.K., Saxena R., Pulikkan J., Grace T., Gomez A., Scaria J., Amato K.R., Sharma V.K. (2019). The unique composition of Indian gut microbiome, gene catalogue, and associated fecal metabolome deciphered using multi-omics approaches. GigaScience.

[60] Serrano J., Smith K.R., Crouch A.L., Sharma V., Yi F., Vargova V., LaMoia T.E., Dupont L.M., Serna V., Tang F. (2021). High-dose saccharin supplementation does not induce gut microbiota changes or glucose intolerance in healthy humans and mice. Microbiome.

[61] Wang T., van Dijk L., Rijnaarts I., Hermes G.D.A., de Roos N.M., Witteman B.J.M., de Wit N.J.W., Govers C., Smidt H., Zoetendal E.G. (2022). Methanogen Levels Are Significantly Associated with Fecal Microbiota Composition and Alpha Diversity in Healthy Adults and Irritable Bowel Syndrome Patients. Microbiol. Spectr..

[62] Li H., Chen J., Ren X., Yang C., Liu S., Bai X., Shan S., Dong X. (2021). Gut Microbiota Composition Changes in Constipated Women of Reproductive Age. Front. Cell. Infect. Microbiol..

[63] Mancabelli L., Milani C., Lugli G.A., Turroni F., Mangifesta M., Viappiani A., Ticinesi A., Nouvenne A., Meschi T., van Sinderen D. (2017). Unveiling the gut microbiota composition and functionality associated with constipation through metagenomic analyses. Sci. Rep..

[64] Rodriguez J., Neyrinck A.M., Zhang Z., Seethaler B., Nazare J.A., Sánchez C.R., Roumain M., Muccioli G.G., Bindels L.B., Cani P.D. (2020). Metabolite profiling reveals the interaction of chitin-glucan with the gut microbiota. Gut Microbes.

[65] Borgo F., Garbossa S., Riva A., Severgnini M., Luigiano C., Benetti A., Pontiroli A.E., Morace G., Borghi E. (2018). Body Mass Index and Sex Affect Diverse Microbial Niches within the Gut. Front. Microbiol..

[66] Gaike A.H., Paul D., Bhute S., Dhotre D.P., Pande P., Upadhyaya S., Reddy Y., Sampath R., Ghosh D., Chandraprabha D. (2020). The Gut Microbial Diversity of Newly Diagnosed Diabetics but Not of Prediabetics Is Significantly Different from That of Healthy Nondiabetics. mSystems.

[67] Ke G., Meng Q., Finley T., Wang T., Chen W., Ma W., Ye Q., Liu T.Y. (2017). LightGBM: A Highly Efficient Gradient Boosting Decision Tree. Adv. Neural Inf. Process. Syst..

[68] Thirion F., Sellebjerg F., Fan Y., Lyu L., Hansen T.H., Pons N., Levenez F., Quinquis B., Stankevic E., Søndergaard H.B. (2023). The gut microbiota in multiple sclerosis varies with disease activity. Genome Med..

[69] Ju T., Kong J.Y., Stothard P., Willing B.P. (2019). Defining the Role of *Parasutterella*, a Previously Uncharacterized Member of the Core Gut Microbiota. ISME J..

[70] Troci A., Zimmermann O., Esser D., Krampitz P., May S., Franke A., Berg D., Leypoldt F., Stürner K.H., Bang C. (2022). B-cell-depletion Reverses Dysbiosis of the Microbiome in Multiple Sclerosis Patients. Sci. Rep..

[71] Moles L., Delgado S., Gorostidi-Aicua M., Sepúlveda L., Alberro A., Iparraguirre L., Suárez J.A., Romarate L., Arruti M., Muñoz-Culla M. (2022). Microbial dysbiosis and lack of SCFA production in a Spanish cohort of patients with multiple sclerosis. Front. Immunol..

[72] Chiodini R.J., Dowd S.E., Chamberlin W.M., Galandiuk S., Davis B., Glassing A. (2015). Microbial Population Differentials between Mucosal and Submucosal Intestinal Tissues in Advanced Crohn’s Disease of the Ileum. PLoS ONE.

[73] Miyake S., Kim S., Suda W., Oshima K., Nakamura M., Matsuoka T., Chihara N., Tomita A., Sato W., Kim S.W. (2015). Dysbiosis in the Gut Microbiota of Patients with Multiple Sclerosis, with a Striking Depletion of Species Belonging to Clostridia XIVa and IV Clusters. PLoS ONE.

[74] Zhang X., Wei Z., Liu Z., Yang W., Huai Y. (2024). Changes in Gut Microbiota in Patients with Multiple Sclerosis Based on 16s rRNA Gene Sequencing Technology: A Review and Meta-Analysis. J. Integr. Neurosci..

[75] Singh V., Lee G., Son H., Koh H., Kim E.S., Unno T., Shin J.H. (2023). Butyrate producers, “The Sentinel of Gut”: Their intestinal significance with and beyond butyrate, and prospective use as microbial therapeutics. Front. Microbiol..

[76] Kibbie J.J., Dillon S.M., Thompson T.A., Purba C.M., McCarter M.D., Wilson C.C. (2021). Butyrate directly decreases human gut lamina propria CD4 T cell function through histone deacetylase (HDAC) inhibition and GPR43 signaling. Immunobiology.

[77] Vacca M., Celano G., Calabrese F.M., Portincasa P., Gobbetti M., De Angelis M. (2020). The Controversial Role of Human Gut *Lachnospiraceae*. Microorganisms.

[78] Letchumanan G., Abdullah N., Marlini M., Baharom N., Lawley B., Omar M.R., Mohideen F.B.S., Addnan F.H., Fariha M.M.N., Ismail Z. (2022). Gut Microbiota Composition in Prediabetes and Newly Diagnosed Type 2 Diabetes: A Systematic Review of Observational Studies. Front. Cell. Infect. Microbiol..

[79] Liu L., Wang H., Chen X., Zhang Y., Zhang H., Xie P. (2023). Gut microbiota and its metabolites in depression: From pathogenesis to treatment. EBioMedicine.

[80] Geng J., Ni Q., Sun W., Li L., Feng X. (2022). The links between gut microbiota and obesity and obesity related diseases. Biomed. Pharmacother..

[81] Chong P.P., Chin V.K., Looi C.Y., Wong W.F., Madhavan P., Yong V.C. (2019). The Microbiome and Irritable Bowel Syndrome—A Review on the Pathophysiology, Current Research and Future Therapy. Front. Microbiol..

[82] Pang R., Wang J., Xiong Y., Liu J., Ma X., Gou X., He X., Cheng C., Wang W., Zheng J. (2022). Relationship between Gut Microbiota and Lymphocyte Subsets in Chinese Han Patients with Spinal Cord Injury. Front. Microbiol..

[83] Kim D.J., Yang J., Seo H., Lee W.H., Lee D.H., Kym S., Park Y.S., Kim J.G., Jang I.J., Kim Y.K. (2020). Colorectal Cancer Diagnostic Model Utilizing Metagenomic and Metabolomic Data of Stool Microbial Extracellular Vesicles. Sci. Rep..

[84] Zeller G., Tap J., Voigt A.Y., Sunagawa S., Kultima J.R., Costea P.I., Amiot A., Böhm J., Brunetti F., Habermann N. (2014). Potential of fecal microbiota for early-stage detection of colorectal cancer. Mol. Syst. Biol..

[85] Chen X., Zhu Z., Zhang W., Wang Y., Wang F., Yang J., Wong K.C. (2022). Human disease prediction from microbiome data by multiple feature fusion and deep learning. iScience.

[86] Oh M., Zhang L. (2020). DeepMicro: Deep representation learning for disease prediction based on microbiome data. Sci. Rep..

[87] Cantoni C., Lin Q., Dorsett Y., Ghezzi L., Liu Z., Pan Y., Chen K., Han Y., Li Z., Xiao H. (2022). Alterations of host-gut microbiome interactions in multiple sclerosis. EBioMedicine.

[88] Tremlett H., Zhu F., Arnold D., Bar-Or A., Bernstein C.N., Bonner C., Forbes J.D., Graham M., Hart J., Knox N.C. (2021). The gut microbiota in pediatric multiple sclerosis and demyelinating syndromes. Ann. Clin. Transl. Neurol..

[89] Montgomery T.L., Wang Q., Mirza A., Dwyer D., Wu Q., Dowling C.A., Martens J.W.S., Yang J., Krementsov D.N., Mao-Draayer Y. (2024). Identification of commensal gut microbiota signatures as predictors of clinical severity and disease progression in multiple sclerosis. Sci. Rep..

[90] Navarro-López V., Méndez-Miralles M.Á., Vela-Yebra R., Fríes-Ramos A., Sánchez-Pellicer P., Ruzafa-Costas B., Núñez-Delegido E., Gómez-Gómez H., Chumillas-Lidón S., Picó-Monllor J.A. (2022). Gut Microbiota as a Potential Predictive Biomarker in Relapsing-Remitting Multiple Sclerosis. Genes.

[91] Ventura R.E., Iizumi T., Battaglia T., Liu M., Perez-Perez G.I., Herbert J., Blaser M.J. (2019). Gut microbiome of treatment-naïve MS patients of different ethnicities early in disease course. Sci. Rep..

[92] Boussamet L., Montassier E., Mathé C., Garcia A., Morille J., Shah S., Dugast E., Wiertlewski S., Gourdel M., Bang C. (2024). Investigating the metabolite signature of an altered oral microbiota as a discriminant factor for multiple sclerosis: A pilot study. Sci. Rep..

[93] Pellizoni F.P., Leite A.Z., Rodrigues N.C., Ubaiz M.J., Gonzaga M.I., Takaoka N.N.C., Mariano V.S., Omori W.P., Pinheiro D.G., Matheucci Junior E. (2021). Detection of Dysbiosis and Increased Intestinal Permeability in Brazilian Patients with Relapsing-Remitting Multiple Sclerosis. Int. J. Environ. Res. Public Health.

[94] Reynders T., Devolder L., Valles-Colomer M., Van Remoortel A., Joossens M., De Keyser J., Nagels G., D’Hooghe M., Raes J. (2020). Gut microbiome variation is associated to Multiple Sclerosis phenotypic subtypes. Ann. Clin. Transl. Neurol..

