# Supplementary material for: Gut Microbiome Signatures in Multiple Sclerosis: A Case-Control Study with Machine Learning and Global Data Integration
Source: Biomedicines. 2025 Jul 23;13(8):1806. doi: 10.3390/biomedicines13081806 (PMC12383397; doi:10.3390/biomedicines13081806)
Supplement: Supplementary file 1 [file biomedicines-13-01806-s001.zip › biomedicines-3647083-supplementary.pdf]

SUPPLEMENTARY FIGURES

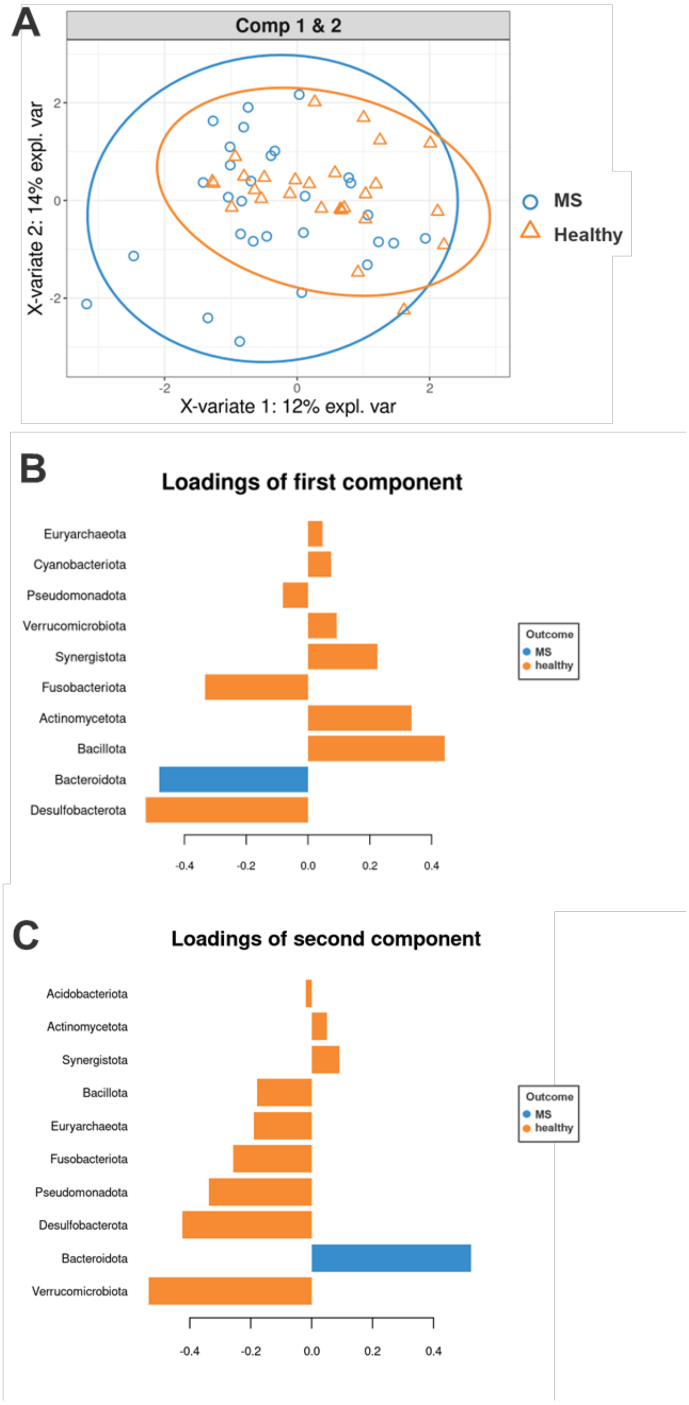

Figure S1: sPLS-DA of the gut microbiota in multiple sclerosis (MS) patients and healthy controls at the phylum level.

(A) sPLS-DA score plot illustrating the separation between healthy controls and MS patients based on gut microbiota composition. Ellipses represent 95% confidence intervals for each group. (B) and (C) show the most discriminative phyla for components 1 and 2, respectively. Taxa are ranked from bottom to top according to their contribution to the corresponding component. Loading weights for healthy controls are shown in orange, while those for MS patients are depicted in blue.

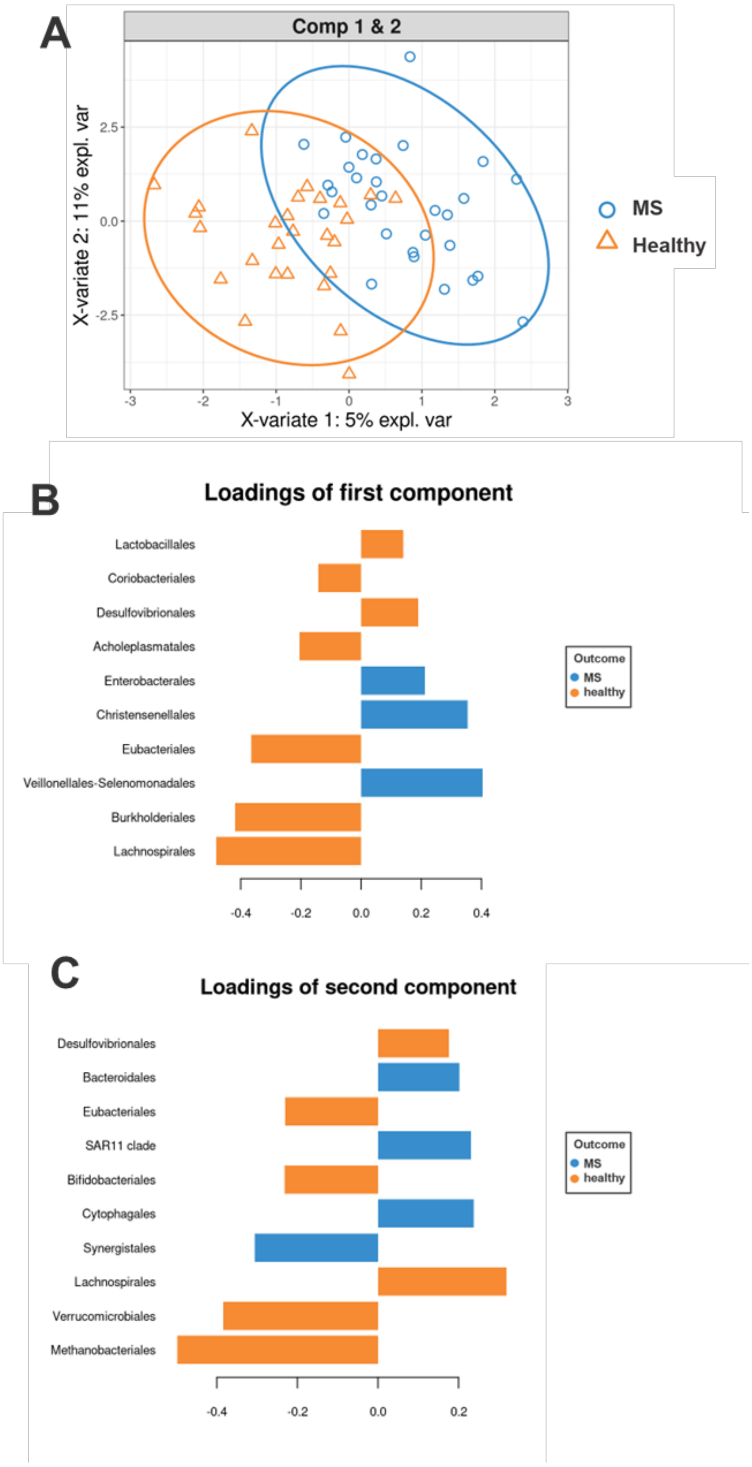

Figure S2: sPLS-DA of the gut microbiota in multiple sclerosis (MS) patients and healthy controls at the order level.

(A) sPLS-DA score plot illustrating the separation between healthy controls and MS patients based on gut microbiota composition. Ellipses represent 95% confidence intervals for each group. (B) and (C) show the most discriminative orders for components 1 and 2, respectively. Taxa are ranked from bottom to top according to their contribution to the corresponding component. Loading weights for healthy controls are shown in orange, while those for MS patients are depicted in blue.

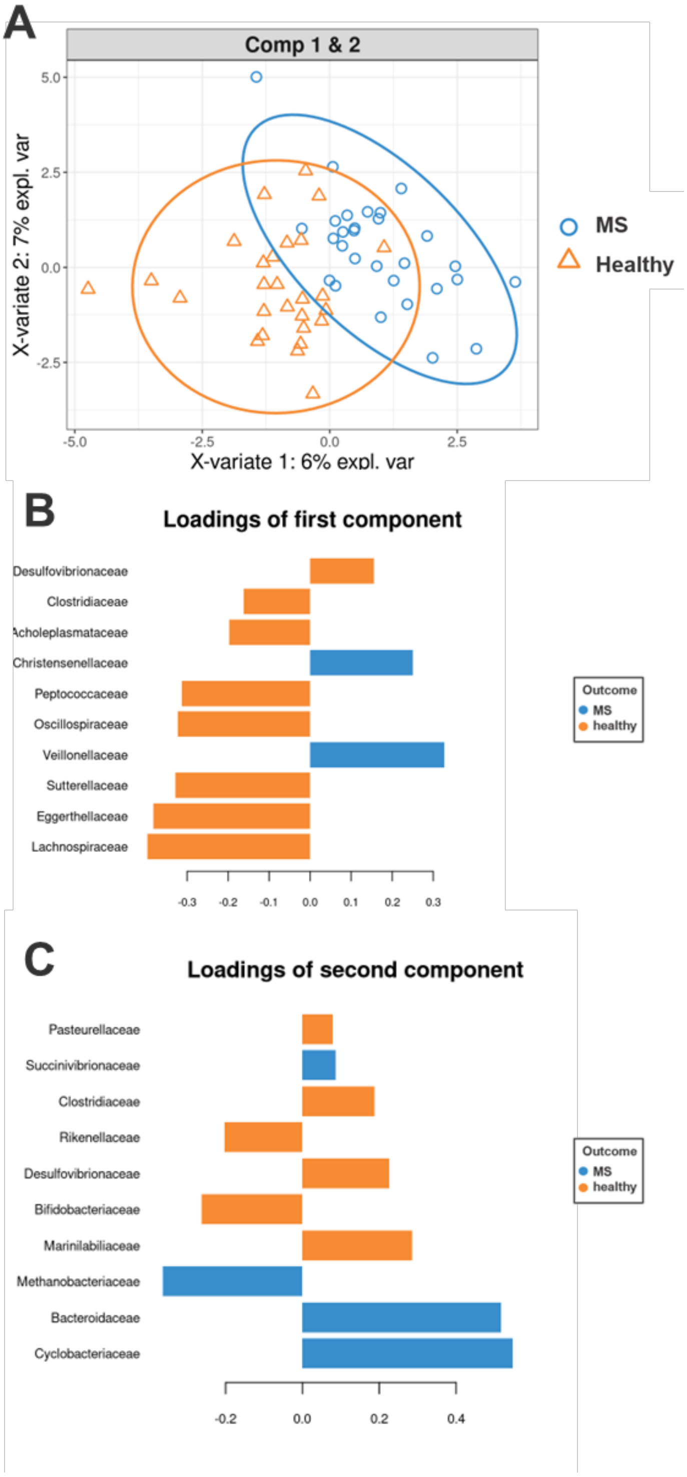

Figure S3: sPLS-DA of the gut microbiota in multiple sclerosis (MS) patients and healthy controls at the family level.

(A) sPLS-DA score plot illustrating the separation between healthy controls and MS patients based on gut microbiota composition. Ellipses represent 95% confidence intervals for each group. (B) and (C) show the most discriminative families for components 1 and 2, respectively. Taxa are ranked from bottom to top according to their contribution to the corresponding component. Loading weights for healthy controls are shown in orange, while those for MS patients are depicted in blue.

SUPPLEMENTARY TABLES

Table S1: Taxa selected for model training as significant for multiple sclerosis (MS). Based on case-control MS studies and results from our study.

№	Taxon name	Reference	Number of studies reporting differences in MS
1	Lachnospiraceae	[87–90]<br>Current study	5
2	*Akkermansia*	[42,43,45,89,91]	5
3	*Roseburia*	[39,45,52,90,92]	5
4	*Faecalibacterium*	[52,73,87]<br>Current study	4
5	*Blautia*	[45,47,52,91]	4
6	*Streptococcus*	[39,45,92,93]	4
7	*Dorea*	[45,52,91]	3
8	*Prevotella*	[42,73,91]	3
9	Oscillospiraceae	[89]<br>Current study	2
10	*Adlercreutzia*	[47,91]	2
11	*Anaerostipes*	[73,87]	2
12	*Bifidobacterium*	[39,93]	2
13	*Lachnospira*	[45,91]	2
14	*Lactobacillus*	[47,92]	2
15	*Methanobrevibacter*	[42,94]	2
16	*Parabacteroides*	[45,93]	2
17	Acidobacteriota	[93]	1
18	*Acinetobacter*	[43]	1
19	*Actinomyces*	[92]	1
20	Bifidobacteriaceae	[93,94]	1

21	*Butyricicoccus*	[94]	1
22	*Butyricimonas*	[42]	1
23	*Collinsella*	[45]	1
24	*Coprococcus*	[45]	1
25	Desulfovibrionaceae	[48]	1
26	Eubacterium ventriosum group	[89]	1
27	*Hungatella*	[90]	1
28	*Porphyromonas*	[90]	1
29	*Pseudomonas*	[47]	1
30	*Ruminococcus*	[90]	1
31	*Sutterella*	[42]	1
32	*Tyzzerella*	[88]	1
33	Eubacteriales	Current study	1
34	Lachnospiraceae UCG-008	Current study	1
35	Lachnospirales	Current study	1
36	*Parasutterella*	Current study	1

Table S2: Light Gradient Boosting Machine model hyperparameters.

Parameter	Value
boosting_type	dart
num_leaves	157
learning_rate	0.38985
n_estimators	759
max_depth	7
subsample	0.584313013
colsample_bytree	0.84586463

reg_alpha	0.43182909
reg_lambda	0.9618589

Table S3: Compositional differences of major taxa in the gut microbiota of multiple sclerosis (MS) patients and healthy controls. The analysis was carried out using the Wilcoxon criterion, with significant differences in taxa highlighted in bold.

	Taxa	MS	Healthy	p-value
**Phylum**	Actinomycetota	0.04 ± 0.005	0.04 ± 0.007	0.66
	Bacillota	0.52 ± 0.013	0.54 ± 0.012	0.11
	Bacteroidota	0.37 ± 0.015	0.35 ± 0.014	0.21
	Pseudomonadota	0.03 ± 0.005	0.03 ± 0.003	0.45
	Verrucomicrobiota	0.02 ± 0.004	0.02 ± 0.005	0.39
**Order**	Bacteroidales	0.46 ± 0.017	0.43 ± 0.017	0.33
	Bifidobacteriales	0.03 ± 0.006	0.04 ± 0.009	0.77
	Erysipelotrichales	0.04 ± 0.007	0.03 ± 0.006	0.92
	**Eubacteriales**	**0.18 ± 0.009**	**0.2 ± 0.008**	**0.04**
	**Lachnospirales**	**0.09 ± 0.005**	**0.12 ± 0.007**	**0.02**
**Family**	Akkermansiaceae	0.02 ± 0.006	0.02 ± 0.008	0.18
	Bacteroidaceae	0.22 ± 0.021	0.19 ± 0.016	0.6
	Barnesiellaceae	0.02 ± 0.004	0.02 ± 0.004	0.78
	Bifidobacteriaceae	0.04 ± 0.007	0.05 ± 0.01	0.84
	Coprobacillaceae	0.03 ± 0.006	0.02 ± 0.004	0.93
	**Lachnospiraceae**	**0.11 ± 0.006**	**0.14 ± 0.008**	**0.01**
	**Oscillospiraceae**	**0.14 ± 0.008**	**0.16 ± 0.007**	**0.01**

	Porphyromonadaceae	0.04 ± 0.004	0.04 ± 0.005	0.43
	Prevotellaceae	0.08 ± 0.019	0.09 ± 0.019	0.92
	Rikenellaceae	0.06 ± 0.009	0.05 ± 0.008	0.65
**Genus**	*Akkermansia*	0.02 ± 0.007	0.03 ± 0.01	0.19
	Alistipes	0.06 ± 0.011	0.06 ± 0.01	0.53
	Bacteroides	0.27 ± 0.024	0.25 ± 0.019	0.63
	Bifidobacterium	0.04 ± 0.007	0.05 ± 0.011	0.6
	Faecalibacterium	**0.06 ± 0.005**	**0.08 ± 0.005**	**0.004**
	**Lachnospiraceae UCG-008**	**0.02 ± 0.001**	**0.02 ± 0.002**	**0.045**
	Parabacteroides	0.02 ± 0.004	0.02 ± 0.004	0.81
	Parasutterella	**0.01 ± 0.003**	**0.02 ± 0.004**	**0.018**
	Porphyromonas	0.05 ± 0.005	0.05 ± 0.006	0.45
	Prevotella	0.04 ± 0.011	0.04 ± 0.015	0.51
	Segatella	0.02 ± 0.006	0.02 ± 0.006	0.54

Table S4: α-diversity analysis (Chao1, Shannon, Pielou indexes) of gut microbiota of multiple sclerosis (MS) patients and healthy controls. Statistical significance was assessed using the Wilcoxon test.

**Taxonomic level**	**α-diversity index**	**MS**	**Healthy**	**p-value**
Phylum	Chao1	14	14	0,78
	Shannon	1,08	1,04	0,97
	Pielou	0,41	0,4	1
Order	Chao1	47	49	0,16

	Shannon	1,9	1,85	0,92
	Pielou	0,48	0,48	0,97
Family	Chao1	78	76	0,72
	Shannon	2,51	2,54	0,63
	Pielou	0,59	0,58	0,58
Genus	Chao1	148	148	0,97
	Shannon	2,93	3	0,77
	Pielou	0,59	0,6	0,94

References for supplementary Table S1

39. Takewaki, D.; Suda, W.; Sato, W.; Takayasu, L.; Kumar, N.; Kimura, K.; Kaga, N.; Mizuno, T.; Miyake, S.; Hattori, M.; Yamamura, T. Alterations of the gut ecological and functional microenvironment in different stages of multiple sclerosis. *Proceedings of the National Academy of Sciences* **2020**, *117*(36), 22402‐22412.

42. Jangi, S.; Gandhi, R.; Cox, L. M.; Li, N.; von Glehn, F.; Yan, R.; Patel, B.; Mazzola, M. A.; Liu, S.; Glanz, B. L.; Cook, S.; Tankou, S.; Stuart, F.; Melo, K.; Nejad, P.; Smith, K.; Topçuolu, B. D.; Holden, J.; Kivisäkk, P.; Chitnis, T.; ⋯ Weiner, H. L. Alterations of the human gut microbiome in multiple sclerosis. *Nat Commun.* **2016**, *7*, 12015.

43. Cekanaviciute, E.; Yoo, B. B.; Runia, T. F.; Debelius, J. W.; Singh, S.; Nelson, C. A.; Kanner, R.; Bencosme, Y.; Lee, Y. K.; Hauser, S. L.; Crabtree-Hartman, E.; Sand, I. K.; Gacias, M.; Zhu, Y.; Casaccia, P.; Cree, B. A. C.; Knight, R.; Mazmanian, S. K.; Baranzini, S. E. Gut bacteria from multiple sclerosis patients modulate human T cells and exacerbate symptoms in mouse models. *PNAS* **2017**, *114*(40), 10713‐10718.

45. Saresella, M.; Marventano, I.; Barone, M.; La Rosa, F.; Piancone, F.; Mendozzi, L.; d'Arma, A.; Rossi, V.; Pugnetti, L.; Roda, G.; Casagni, E.; Cas, M. D.; Paroni, R.; Brigidi, P.; Turroni, S.; Clerici, M. Alterations in Circulating Fatty Acid Are Associated With Gut Microbiota Dysbiosis and Inflammation in Multiple Sclerosis. *Front Immunol.* **2020**, *11*, 1390.

47. Chen, J.; Chia, N.; Kalari, K. R.; Yao, J. Z.; Novotna, M.; Paz Soldan, M. M.; Luckey, D. H.; Marietta, E. V.; Jeraldo, P. R.; Chen, X.; Weinshenker, B. G.; Rodriguez, M.; Kantarci, O. H.; Nelson, H.; Murray, J. A.; Mangalam, A. K. Multiple sclerosis patients have a distinct gut microbiota compared to healthy controls. *Sci Rep.* **2016**, *6*, 28484.

48. Kozhieva, M.; Naumova, N.; Alikina, T.; Boyko, A.; Vlassov, V.; Kabilov, M. R. Primary progressive multiple sclerosis in a Russian cohort: relationship with gut bacterial diversity. *BMC microbiol.* **2019**, *19*(1), 309.

52. Ling, Z.; Cheng, Y.; Yan, X.; Shao, L.; Liu, X.; Zhou, D.; Zhang, L.; Yu, K.; Zhao, L. Alterations of the Fecal Microbiota in Chinese Patients With Multiple Sclerosis. *Front Immunol.* **2020**, *11*, 590783.

73. Miyake, S.; Kim, S.; Suda, W.; Oshima, K.; Nakamura, M.; Matsuoka, T.; Chihara, N.; Tomita, A.; Sato, W.; Kim, S. W.; Morita, H.; Hattori, M.; Yamamura, T. Dysbiosis in the Gut Microbiota of Patients with Multiple Sclerosis, with a Striking Depletion of Species Belonging to Clostridia XIVa and IV Clusters. *PloS one* **2015**, *10*(9), e0137429.

87. Cantoni, C.; Lin, Q.; Dorsett, Y.; Ghezzi, L.; Liu, Z.; Pan, Y.; Chen, K.; Han, Y.; Li, Z.; Xiao, H.; Gormley, M.; Liu, Y.; Bokoliya, S.; Panier, H.; Suther, C.; Evans, E.; Deng, L.; Locca, A.; Mikesell, R.; Obert, K.; … Zhou, Y. Alterations of host-gut microbiome interactions in multiple sclerosis. *EBioMedicine* **2022**, *76*, 103798.

88. Tremlett, H.; Zhu, F.; Arnold, D.; Bar-Or, A.; Bernstein, C. N.; Bonner, C.; Forbes, J. D.; Graham, M.; Hart, J.; Knox, N. C.; Marrie, R. A.; Mirza, A. I.; O'Mahony, J.; Van Domselaar, G.; Yeh, E. A.; Zhao, Y.; Banwell, B.; Waubant, E.; US Network of Pediatric MS Centers, the Canadian Pediatric Demyelinating Disease Network. The gut microbiota in pediatric multiple sclerosis and demyelinating syndromes. *Ann Clin Transl Neurol.* **2021**, *8*(12), 2252-2269.

89. Montgomery, T. L.; Wang, Q.; Mirza, A.; Dwyer, D.; Wu, Q.; Dowling, C. A.; Martens, J. W. S.; Yang, J.; Krementsov, D. N.; Mao-Draayer, Y. Identification of commensal gut microbiota signatures as predictors of clinical severity and disease progression in multiple sclerosis. *Sci Rep.* **2024**, *14*(1), 15292.

90. Navarro-López, V.; Méndez-Miralles, M. Á.; Vela-Yebra, R.; Fríes-Ramos, A.; Sánchez-Pellicer, P.; Ruzafa-Costas, B.; Núñez-Delegido, E.; Gómez-Gómez, H.; Chumillas-Lidón, S.; Picó-Monllor, J. A.; Navarro-Moratalla, L. Gut Microbiota as a Potential Predictive Biomarker in Relapsing-Remitting Multiple Sclerosis. *Genes* **2022**, *13*(5), 930.

91. Ventura, R. E.; Iizumi, T.; Battaglia, T.; Liu, M.; Perez-Perez, G. I.; Herbert, J.; Blaser, M. J. Gut microbiome of treatment-naïve MS patients of different ethnicities early in disease course. *Sci Rep.* **2019**, *9*(1), 16396.

92. Boussamet, L.; Montassier, E.; Mathé, C.; Garcia, A.; Morille, J.; Shah, S.; Dugast, E.; Wiertlewski, S.; Gourdel, M.; Bang, C.; Stürner, K. H.; Masson, D.; Nicot, A. B.; Vince, N.; Laplaud, D. A.; Feinstein, D. L.; Berthelot, L. Investigating the metabolite signature of an altered oral microbiota as a discriminant factor for multiple sclerosis: a pilot study. *Sci Rep*. **2024**, *14*(1), 7786.

93. Pellizoni, F. P.; Leite, A. Z.; Rodrigues, N. C.; Ubaiz, M. J.; Gonzaga, M. I.; Takaoka, N. N. C.; Mariano, V. S.; Omori, W. P.; Pinheiro, D. G.; Matheucci Junior, E.; Gomes, E.; de Oliveira, G. L. V. Detection of Dysbiosis and Increased Intestinal Permeability in Brazilian Patients with Relapsing-Remitting Multiple Sclerosis. *Int J Environ Res Public Health*. **2021**, *18*(9), 4621.

94. Reynders, T.; Devolder, L.; Valles-Colomer, M.; Van Remoortel, A.; Joossens, M.; De Keyser, J.; Nagels, G.; D'hooghe, M.; Raes, J. Gut microbiome variation is associated to Multiple Sclerosis phenotypic subtypes. *Ann Clin Transl Neurol*. **2020**, *7*(4), 406-419.
